# Application of MAT Methodology in the Evaluation of Prescribing Adherence to Clinical Practice Guidelines for Secondary Prevention of Coronary Heart Disease in Post-Acute Coronary Syndrome Patients in Kuwait

**DOI:** 10.3389/fphar.2021.647674

**Published:** 2021-10-04

**Authors:** Dalal Al-Taweel, Abdelmoneim Awad

**Affiliations:** Department of Pharmacy Practice, Faculty of Pharmacy, Kuwait University, Kuwait City, Kuwait

**Keywords:** medication adherence, guideline adherence, ST Elevation Myocardial Infarction, Non ST Elevation Myocardial Infarction, practice guideline

## Abstract

Quantification of prescribers’ adherence to evidence-based guidelines can be used as an outcome measure to assess the impact of services on the quality of medication use. Additionally, it can help in reducing inappropriate interventions and ensure that high-quality care is provided to patients. This study aimed to evaluate prescribing practices for secondary prevention of coronary heart disease (CHD) in post-acute coronary syndromes (ST-elevation myocardial infarction [STEMI] or non-ST elevation acute coronary syndrome [NSTEACS]) patients using two medication assessment tools (MATs) at secondary and tertiary health-care settings in Kuwait. Both MATs were developed and validated based on the relevant guidelines issued by the European Society of Cardiology and the American College of Cardiology/American Heart Association. A quantitative cross-sectional multicenter study was conducted on 460 patients’ medical records collected randomly from six health-care facilities in Kuwait. Application of MAT_STEMI_ on 232 patients’ medication records (with 85.9% applicability) resulted in intermediate overall adherence (69.8%; 95% CI: 67.6–72.0). Application of MAT_NSTEACS_ on 228 patients’ medication records (with applicability 83.2%) resulted in intermediate overall adherence (73.3%; 95% CI: 70.5–76.0). There was no significant difference between the percentages of overall adherence among patients managed post-NSTEACS compared to those managed post-STEMI (*p* = 0.05). Multivariable logistic regression analysis revealed that the overall adherence to the MAT_STEMI_ criteria was significantly higher among the specialized cardiac centers than among the general hospitals (OR: 1.6; 95% CI: 1.1–2.3; *p* = 0.02). The overall adherence to the MAT_NSTEACS_ criteria was found to be significantly lower among non-Kuwaitis than among Kuwaitis (OR: 0.6; 95% CI: 0.5–0.9; *p* = 0.01) and patients with a serum LDL ≥1.8 mmol/L than those with a serum LDL-C < 1.8 mmol/L (OR: 0.5; 95% CI: 0.4–0.7; *p* < 0.001). The present findings revealed that both MATs were useful tools in identifying the standard of clinical performances and highlighting areas for improvement regarding secondary prevention of CHD in post-acute coronary syndrome patients.

## Introduction

The prevention and management of chronic cardiovascular disorders have been reported as a global health priority since cardiovascular diseases (CVDs) are proven to be the leading cause of mortality worldwide (WHO, 2018). CVDs include coronary heart disease (CHD), cerebrovascular disease, peripheral arterial disease, rheumatic heart disease, congenital heart disease, deep vein thrombosis, and pulmonary embolism (WHO, 2018). As with many chronic diseases, applying evidence behind CVD management into practice often proves to be difficult as health-care providers find themselves managing between benefits and risks to individual patients, patients’ health preferences, as well as evidence-based medicine when managing patients with multiple disease states (Dreischulte et al., 2013; Erhardt, 2005; Steinman, 2007). Previously published research has stated that successful adoption of clinical practice guidelines (CPGs) in the management of CVDs can have a positive impact on many factors including prescribers’ involvement in care, patient outcome expectancy, and prescribing habits and routines, in addition to administrative factors (Mosca et al., 2005). CPGs are designed to synthesize and disseminate the best available evidence to guide clinical practice (Ryan, 2017). They have proven to be of value in standardizing care to patients as they allow practitioners to provide systematic care to their patients that is evidence-based (Woolf et al., 1999). Quantification of prescribers’ adherence to CPGs serves as an outcome measure to evaluate the influence of services on the quality of medication use. Additionally, it can help in reducing inappropriate interventions and ensure that high-quality care is provided to patients. Although evidence has shown positive effects of adherence to CPGs on patients’ health-care outcomes (Lugtenberg et al., 2009; Arabi et al., 2010) and cost-effectiveness (Davis and Taylor-Vaisey, 1997; Klazinga, 2003), studies have shown that many barriers exist that prevent prescribers from adhering to CPGs.

Medication assessment tools (MATs) have been designed as clinical audit tools to measure prescribing adherence of clinicians to set medication use standards by applying them retrospectively to patients’ medical records. Globally, they have been used to detect opportunities to enhance medication therapy management in many chronic diseases and identify deviations from best practice (Hakonsen et al., 2006; Hakonsen et al., 2009; McAnaw and McGlynn, 2003; Kamyar et al., 2008; Al-Taweel et al., 2013; Diab et al., 2013). Adherence of prescribers to CPGs is reported with the aim to achieve optimal care at a minimal cost of application and a higher degree of objectivity (Kaufmann et al., 2014). Nevertheless, it is important to review and update these tools regularly to reflect the most up-to-date evidence and recommendations.

Medication therapy standards and therapeutic goals, in CHD, are well-defined in published evidence-based guidelines; however, prescribing practices in the management of this disease state are still suboptimal (Kotseva et al., 2009). Therefore, the development of MATs through the extraction of medication-related criteria to check adherence to recommendations from CPGs is a potential solution for optimizing patient care by detecting gaps in the prescribing practice in the management of secondary prevention of CHD. Evidence has shown that adhering to quality standards published in clinical practice guidelines results in improving patient outcomes and reducing mortality rates (Kuepper-Nybelen et al., 2012; Mukherjee et al., 2004)*.* Limited studies have been conducted in the developed countries using the MAT methodology to evaluate the management of secondary prevention of CHD (Kamyar et al., 2008; Garcia et al., 2011; Garcia et al., 2013)*.* However, there are no similar published studies from the developing countries including the Middle East and North Africa (MENA) region. To the best of our knowledge, there is only one published study that reported the development and validation of MATs to evaluate prescribing adherence to CPGs for secondary prevention of CHD in post-acute coronary syndrome patients in Kuwait. Its findings explicitly defined the criteria in both MATs and reported the face and content validity. Also, the feasibility testing of both MATs was performed on 66 patients’ medical records as a pilot study. The results revealed an intermediate overall adherence to the MAT_STEMI_ (64.1%) and MAT_NSTEACS_ (62.0%) (Al-Taweel and Awad, 2020)*.*

In Kuwait, there has been an increased interest in the management of patients with CVD and the provision of evidence-based clinical services by practitioners in recent years (Zubaid et al., 2020; Al-Zakwani et al., 2018; Longenecker et al., 2013). However, there is a lack of the literature assessing prescribing practices in the management of CVDs by health-care providers. This information is needed as a basis for the development of a quality assurance framework aimed at ensuring the best possible patient care. This is a multicenter study that aimed to evaluate the quality of prescribing practices for secondary prevention of CHD in post-acute coronary syndrome (ST-elevation myocardial infarction [STEMI] or non-ST elevation acute coronary syndrome [NSTEACS including NSTEMI and unstable angina]) patients attending secondary and tertiary health-care settings. Two developed and validated MATs derived from the ESC and ACC/AHA CPGs were used for the evaluation of the prescribing practices.

## Materials and Methods

### Study Area

Kuwait is a country in the Middle East, situated between Saudi Arabia and Iraq, with an area of 17,820 km^2^ and an estimated total population of 4,334,391 people (2021 estimates). Expatriates (non-Kuwaiti residents) account for about 70%, including 1.1 million Arab expatriates and 1.4 million Asian expatriates (World Population Review, 2021). Health care in Kuwait is provided through a public and a private sector, with the largest provider being the public sector. Health care is provided free of charge for Kuwaiti citizens and at a reduced cost for non-Kuwaiti residents who are enrolled in a public insurance scheme. The private sector accepts all residents in Kuwait whether they have private health insurance or are uninsured. The public health-care sector is divided into primary, secondary, and tertiary settings. Primary health-care settings are often the first point of care for residents, with general practitioners or family medicine specialists providing a variety of services including chronic disease clinics, maternity clinics, preventive care clinics, and dental clinics. Secondary health-care settings provide more advanced services at six public hospitals equipped with outpatient clinics and a 24-h casualty service. More specialized care is provided through tertiary health-care settings, such as a cardiac center, a cancer control center, a speech and swallowing center, transplant centers, and dermatology centers that focus on specific conditions.

### Study Design

A quantitative, cross-sectional study was performed to evaluate the quality of prescribing at secondary and tertiary care settings of patients diagnosed with post-acute coronary syndromes (STEMI or NSTEACS including NSTEMI and unstable angina) in Kuwait. The study was conducted between January 2019 and January 2020. Ethical approval for the present study was obtained from the Medical Research Ethics Committee of the Ministry of Health and the Human Ethical Committee, Health Sciences Centre, Kuwait University (Ethics No: 2018/866). Informed consent was not required as data were collected from patients’ medical records retrospectively. Data were extracted anonymously as directly identifying patient information was not obtained.

### Inclusion and Exclusion Criteria

Criteria for inclusion involved patients aged 18–75 years, who are currently alive, and who had experienced either STEMI or NSTEACS within at least 12 months before the study period and had attended the cardiovascular outpatient clinics for the long-term management of STEMI or NSTEACS during the past 2 years before the study period. Patients who had not attended the clinic within the last 2 years prior to the study period and patients who are pregnant or breastfeeding were excluded from the study sample. Also, patients aged <18 years or >75 years were excluded because more cautious goals are often used for these populations. Any incomplete data were reported as “missing data” and was sub-reported.

### Study Sample

The sample size is based on the assumption that the proportion of adherence to clinical guidelines for the treatment of CVDs is 50% as there are no preceding studies from Kuwait or other Middle Eastern countries that are similar to our current study. The Raosoft sample size calculator was used to calculate the sample size using a margin of error of 5%, a confidence interval of 95%, a population size of about 200,000 patients with CVDs, and an expected adherence of 50%. The minimum sample size estimated for the study was 384 (Raosoft, 2004). Assuming complete patients’ medical records with sufficient data of 85%, a larger sample size of 460 patients’ medical records was collected from six health-care facilities (3 general hospitals with cardiology units and 3 specialized cardiac centers). A total of 76–77 patients’ medical records were collected from the outpatient clinics in each health-care facility using stratified and systematic random sampling.

### Study Tool

Two MATs (MAT_STEMI_ and MAT_NSTEACS_) were developed and validated in an earlier study by the same authors (Al-Taweel and Awad, 2020). Recommendations from European and American guidelines, the European Society of Cardiology (ESC) guidelines (Roffi et al., 2016; Ibanez et al., 2018) and the American College of Cardiology/American Heart Association (ACC/AHA) guidelines (Amsterdam et al., 2014; O'Gara et al., 2013), were used to design the MATs. This was based on the guidelines used at the time of the study by cardiologists in the secondary prevention of CHD in Kuwait. A total of 16 criteria for MAT_STEMI_ and 11 criteria for MAT_NSTEACS_ were developed from these guidelines. Each MAT criterion (e.g., the patient with no contraindication to aspirin is prescribed a daily dose of aspirin 81–325 mg, indefinitely) is in the form of two statements—a qualifying statement (the patient with no contraindication to aspirin) that specifies patient conditions under which a guideline-recommended treatment, represented by the standard statement (is prescribed a daily dose of aspirin 81–325 mg, indefinitely), applies. The qualifying statement (the qualifier, in bold font, as seen in Tables 1, 2) assessed the applicability of each patient to each criterion. The standard was only considered when the qualifier was applicable to the patient and reflected the expectation of the criterion. Not applicable (NA) was labeled if the patient did not meet the qualifier, which reflects that the patient was not eligible for the application of the criterion. An example of a criterion for post-STEMI patients is “the patient post-fibrinolysis without subsequent PCI is prescribed clopidogrel 75 mg daily in addition to aspirin for at least 14 days and up to 1 year in absence of bleeding.” The criterion was labeled “NA” if the patient had not been given a fibrinolytic or was given a fibrinolytic with subsequent PCI. For the applicable criteria, each was assigned one of five answer categories: 1) yes—the standard was adhered to in the eligible patients (i.e., the patient met both the qualifier and standard; 2) no (J)—the standard is not adhered to in the eligible patients (i.e., the patient met the qualifier but not the standard), but a justification was documented in the patient’s medical notes; 3) no (U)—the standard is not adhered to in the eligible patients (i.e., the patient met the qualifier but not the standard), and there is no apparent reason or justification documented in the patient’s medical records; 4) IDQ—insufficient data (i.e., lack of information) on the qualifier to assess whether a criterion was applicable or not; and 5) IDS—insufficient data (i.e., lack of information) on the standard to assess whether the applicable criterion met the standard statement or not.

**TABLE 1:** Adherence to the criteria of MAT_STEMI_ (*n* = 232).

No	Criteria^a^	Applicability	% Adjusted Adherence (95%CI)
**Antithrombotic therapy**			
1	**Patient with no contraindication to aspirin** is prescribed a daily dose of aspirin 81–325 mg, indefinitely	230	90.4 (85.7–93.8)
2	**Patient who is not prescribed aspirin due to hypersensitivity** is prescribed clopidogrel 75 mg daily	23	100 (82.2–99.6)
3	**Patient with stent post-primary PCI** is prescribed clopidogrel 75 mg or ticagrelor 90 mg BID daily for at least 12 months, in addition to aspirin 81 mg as a dual therapy	135	77.0 (68.9–83.7)
4	**Patient post-fibrinolysis without subsequent PCI** is prescribed clopidogrel 75 mg daily in addition to aspirin for at least 14 days and up to 1 year in absence of bleeding	8	37.5 (10.2–74.1)
5	**Patient post-fibrinolysis with subsequent PCI** is prescribed clopidogrel 75 mg daily in addition to aspirin for 12 months	64	90.6 (80.1–96.1)
6	**Patient on dual antiplatelet therapy and at higher than average risk of gastrointestinal bleeding** is prescribed a proton pump inhibitor	23	82.6 (60.5–94.3)
**Beta-blockers**			
7	**Patient with no contraindications to beta-blockers** is prescribed a beta-blocker	231	93.9 (89.82–96.5)
8	**Patient with no contraindications to beta-blockers and prescribed a beta-blocker** is prescribed metoprolol succinate SR, bisoprolol, or carvedilol for up to 3 years	160	92.5 (87.0–95.9)
9	**Patient with no contraindications to beta-blockers with an LVEF ≤ 40% and prescribed a beta-blocker** is prescribed either a metoprolol succinate SR, bisoprolol, or carvedilol indefinitely	61	93.4 (83.3–97.9)
**Lipid-lowering therapies**			
10	**Patient regardless of the lipid level** is prescribed a high intensity statin either atorvastatin 40–80 mg or rosuvastatin 20–40 mg	232	45.7 (39.2–52.3)
11	**Patient prescribed a high intensity statin** is prescribed atorvastatin 80 mg	104	22.1 (14.8–31.5)
12	**Patient maintained on statins with a baseline LDL level 1.8–3.5 mmol/L** has achieved a target LDL cholesterol <1.8 mmol/L or at least 50% reduction in LDL cholesterol	231	48.9 (42.3–55.5
13	**Patient with an LDL ≥ 1.8 mmol/L and despite a maximally tolerated statin** should be on further therapy (ezetimibe)	79	10.1 (4.8–19.5)
**Inhibitors of renin–angiotensin–aldosterone system**			
14	**Patient with no contraindication to ACE inhibitors** is prescribed an ACE inhibitor	191	91.6 (86.5–95.0)
15	**Patient not prescribed ACE inhibitor due to intolerance** is prescribed ARB	33	100 (87.0–99.7)
16	**Patient already receiving an ACEI and beta-blocker and have LVEF ≤ 40%, and either heart failure or diabetes (without significant renal dysfunction, or hyperkalemia)** is prescribed an aldosterone antagonist	55	54.6 (40.7–67.8)

a Criteria were developed and validated from the ESC guidelines (Roffi et al., 2016; Ibanez et al., 2018) and the ACC/AHA guidelines (Amsterdam et al., 2014; O'Gara et al., 2013).

**TABLE 2:** Adherence to the criteria of MAT_NSTEACS_ (*n* = 228).

No	Criteria^a^	Applicability	% Adjusted adherence (95%CI)
**Antithrombotic therapy**			
1	**Patient with no contraindication to aspirin** is prescribed a daily dose of aspirin 81–325 mg, indefinitely	228	95.6 (91.8–97.8)
2	**Patient who is not prescribed aspirin due to hypersensitivity** is prescribed clopidogrel 75 mg daily	6	100 (51.7–98.5)
3	Patient treated with the ischemic-guided strategy, in addition to aspirin 81 mg (if not contraindicated), is prescribed clopidogrel 75 mg OD or ticagrelor 90 mg BID for a duration of up to 12 months	46	54.3 (39.2–68.8)
4	**Patient with stent post primary PCI** is prescribed clopidogrel 75 mg or ticagrelor 90 mg BID daily for at least 12 months, in addition to aspirin 81 mg as a dual therapy	163	86.5 (80.1–91.2)
5	**Patient on dual antiplatelet therapy and at higher than average risk of gastrointestinal bleeding** is prescribed a proton pump inhibitor	40	90.0 (75.4–96.8)
**Beta-blockers**			
6	**Patient with an LVEF ≤ 40% with no contraindications to beta-blockers and prescribed a beta-blocker** is prescribed either metoprolol succinate SR, bisoprolol, or carvedilol	38	92.1 (77.5–97.9)
**Lipid-lowering therapies**			
7	**Patient regardless of lipid levels** is prescribed a high intensity statin either atorvastatin 40–80 mg or rosuvastatin 20–40 mg	228	61.0 (54.3–67.3)
8	**Patient with an LDL ≥ 1.8 mmol/L and despite a maximally tolerated statin** should be on further therapy (ezetimibe)	92	13.0 (7.2–22.1)
**Inhibitors of renin–angiotensin–aldosterone system**			
9	**Patient with a confirmed LVEF ≤ 40% or heart failure, or hypertension or diabetes** is prescribed an ACE inhibitor	199	89.5 (84.1–93.2)
10	**Patient with intolerance to ACE inhibitors with a confirmed LVEF ≤ 40% or heart failure, or hypertension or diabetes** is prescribed ARB	28	100 (85.0–99.7)
11	**Patient already receiving an ACEI and beta-blocker and have LVEF ≤ 40%, and either heart failure or diabetes (without significant renal dysfunction, or hyperkalemia)** is prescribed an aldosterone antagonist	37	29.7 (16.4–47.2)

a Criteria were developed and validated from the ESC guidelines (Roffi et al., 2016; Ibanez et al., 2018) and the ACC/AHA guidelines (Amsterdam et al., 2014; O'Gara et al., 2013).

### Data Collection and Statistical Analysis

Data were collected from the outpatient clinics in each health-care facility. Data were extracted from the selected patients’ medical records using standard predefined data collection forms. The authors of this study, both pharmacists, reviewed the data thoroughly based on MAT_STEMI_ and MAT_NSTEACS_ criteria. Discrepancies were discussed until a consensus was reached. The first author has a Ph.D. in pharmaceutical care with experience in MAT development, and the second author is a professor of clinical pharmacy with experience in cardiovascular and geriatric pharmacotherapy. The Statistical Package for Social Sciences (IBM SPSS Statistics for Windows, version 25, Armonk, NY: IBM Corp) was used to analyze the data. The normality of data distribution was tested using the Shapiro–Wilk and Kolmogorov–Smirnov tests, which revealed that the data were not normally distributed. The results were presented as percentages (95% confidence intervals; CI), medians (interquartile range; IQR), and means (standard deviation; SD). The percentages of unadjusted adherence and adjusted adherence to each criterion, as well as the overall adherence for each MAT, were calculated as follows: The percentage of unadjusted adherence was calculated from the summation of all adhered criteria (criteria recorded “Yes” [numerator]) over the summation of all applicable criteria (criteria recorded “Yes,” “No(U),” and “IDS” [denominator]) “[% Adherence = [Σ Yes]/[Σ Yes + No(U)+IDS)] x 100].” The percentage of adjusted adherence was calculated from the summation of all adhered criteria (criteria recorded “Yes” + “No(J)” [numerator]) over the summation of all applicable criteria (criteria recorded “Yes,” “No(U),” “No(J),” and “IDS” [denominator]) “[% Adjusted adherence = [Σ Yes + No(J)]/(Σ Yes + No(U)+No(J)+IDS] x 100].” It is important to calculate % adjusted adherence as it is the true adherence by taking into account that No(J) is not a “non-adherence” since there is a justification, and the physician is aware that he/she is not adhering. Hence, only the results of adjusted adherence are reported. The overall % non-adherence = [Σ No(J) + No(U)]/[ Σ Yes + IDS + No(U) + No(J)] x 100 was also calculated. The overall adherence and non-adherence were calculated after the deletion of criteria with very low applicability (less than 10 patients). Also, the overall % data gap [IDS/(Yes + No(U)+No(J)+IDS] x 100] was calculated as it gives a representation of how well data are documented. The level of adherence to the criteria was ranked based on cutoffs used in a previous study of similar design (high, ≥ 80%; intermediate, 50 to <80%; low <50%) (Al-Taweel et al., 2013).

Comparison of characteristics between patients diagnosed with prior STEMI and NSTEACS was carried out using the chi-square and Mann–Whitney test. Statistical significance is considered at a level of *p* < 0.05. Univariable logistic regression was performed to determine the association of patients’ characteristics (health-care facilities, age, sex, nationality, smoking, duration since diagnosis, past medical history (PMH) of stable ischemic heart disease, PMH of hypertension, PMH of dyslipidemia, PMH of diabetes, left ventricular ejection fraction, BP, and serum LDL-C) with the dependent variables (overall adherence to the MAT_STEMI_ criteria and MAT_NSTEACS_ criteria). All variables with *p* ≤ 0.25 in the univariable analysis were included in the multiple logistic regression analysis to determine the factors that are associated with each dependent variable. Only the results of the multivariable logistic analysis are reported showing the odds ratio (OR) and 95% CI.

## Results

### Demographics of the Study Population

Table 3 presents the characteristics of patients diagnosed with prior STEMI and prior NSTEACS included in the study. Of the 460 patients, 50.4% and 49.6% were diagnosed with prior STEMI and prior NESTEACS, respectively. Their overall results revealed that their median (IQR) age was 57.0 (16) years [mean (SD): 57.5 (10.9)], 76.1% were diagnosed during the last 5 years before the study period, 74.8% were female, and 52.2% were non-Kuwaitis. Almost two-fifths (*n* = 177, 38.5%) of the patients were current non-smokers, of whom 71 (40.1%) were ex-smokers. The median (IQR) age [59 (16) vs 54 (17.0)] and duration since diagnosis [3 (4) vs 2 (4)] were significantly higher among patients with prior NSTEACS than those with prior STEMI (*p* = 0.003 and <0.001, respectively). Males and non-Kuwaitis were significantly more among patients with prior STEMI than those with prior NSTEACS (*p* = 0.009 and *p* = 0.01, respectively). Female individuals and Kuwaitis were significantly more among patients with prior NSTEACS than those with prior STEMI (*p* = 0.009).

**TABLE 3:** Demographic and other characteristics of patients (*n* = 460).

	Post-STEMI, *n* (%)	Post-NSTEACS, *n* (%)	Total, *n* (%)
**Patients**	232 (50.4)	228 (49.6)	460 (100)
**Duration since diagnosis (years)**			
1–5	187 (80.6)	163 (71.5)	350 (76.1)
6–10	30 (12.9)	32 (14.0)	62 (13.5)
≥10	12 (5.2%)	29 (12.7)	41 (8.9)
Missing	3 (1.3%)	4 (1.8)	7 1.5)
**Health-care facilities**			
General hospitals	126 (54.3)	106 (46.5)	232 (50.4)
Specialized cardiac centers	106 (45.7)	122 (53.5)	228 (49.6)
**Sex**			
Male	186 (80.2)	158 (69.3)	344 (74.8)
Female	44 (19.0)	68 (29.8)	112 (24.4)
Missing	2 (0.8)	2 (0.9)	4 (0.8)
**Age (years)**			
20–44	31 (13.4)	23 (10.1)	54 (11.7)
45–64	142 (61.2)	128 (56.1)	270 (58.7)
≥65	59 (25.4)	77 (33.8)	136 (29.6)
**Nationality**			
Kuwaiti	91 (39.2)	118 (51.7)	209 (45.4)
Non-Kuwaiti	135 (58.2)	105 (46.1)	240 (52.2)
Missing	6 (2.6)	5 (2.2)	11 (2.4)
**Current smokers**			
Yes	64 (27.6)	49 (21.5)	113 (24.6)
No	94 (40.5)	83 (36.4)	177 (38.5)
Missing	74 (31.9)	96 (42.1)	170 (36.9)

Of the 232 patients managed post-STEMI, 21 (9.1%; 95% CI: 5.8–13.7) were treated with fibrinolysis without subsequent percutaneous coronary intervention (PCI) and stent, 65 (28.0%; 95% CI: 22.4–34.3) were treated with fibrinolysis with subsequent PCI and stent, and 146 (62.9%; 95% CI: 56.3–69.1) were treated with PCI and stent. On the other hand, of the 228 patients managed post-NSTEACS, 61 (26.7%; 95% CI: 21.2–33.1) were treated with ischemic guided strategy and 167 (73.3%; 95% CI: 66.9–78.8) were treated with PCI and stent. Over two-thirds of patients had a PMH of hypertension (68.7%) and stable ischemic heart disease (67.4%), 51.7% had dyslipidemia, 50.6% had diabetes, and 21.5% with a left ventricular ejection fraction (LVEF) of ≤40%. Of the available clinical data, the percentages of patients who had reached the target BP and lipid profile were as follows: 179 out of 409 (43.8%) had a BP < 130/80 mmHg, 122 out of 289 (42.2%) had an LDL <1.8 mmol/L, 138 out of 290 (47.6%) had a non-HDL < 2.59 mmol/L, and 182 out of 296 (61.5%) had triglycerides <1.7 mmol/L. The percentages of patients who had PMH of hypertension (75.4 vs 62.1%) or diabetes (56.1 vs 45.2%) were significantly higher among patients with prior NSTEACS than those with prior STEMI (*p* = 0.002 and *p* = 0.2, respectively). The percentage of patients who had an LVEF of ≤40 was significantly higher among patients with prior STEMI (26.3%) than those with prior NSTEACS (16.7%); *p* = 0.02. There were no significant differences between both groups of patients regarding the other characteristics (*p* > 0.05). Table 4 presents the clinical characteristics of the patients.

**TABLE 4:** Clinical characteristics of patients (*n* = 460).

Characteristic	Post-STEMI (*n* = 232)	Post-NSTEACS (*n* = 228)	Total, n (%)
**PMH of CVD and/or major CVD risk factors, *n* (%)**			
Stroke/TIA	11 (4.7)	12 (5.3)	23 (5.0)
Stable ischemic heart disease	151 (65.1)	159 (68.5)	310 (67.4)
Hypertension	144 (62.1)	172 (75.4)	316 (68.7)
Dyslipidemia	110 (47.4)	128 (42.4)	238 (51.7)
Diabetes	105 (45.3)	128 (42.4)	233 (50.6)
**Left ventricular ejection fraction (%)**			
≤40 *n* (%)	61 (26.3)	38 (16.7)	99 (21.5)
41–49 *n* (%)	36 (15.5)	28 (12.3)	64 (13.9)
≥50 *n* (%)	123 (53.0)	151 (66.2)	274 (59.6)
Missing, *n* (%)	12 (5.2)	11 (4.8)	23 (5.0)
**BP (mmHg)**			
SBP and DBP <130/80 *n* (%)	95 (40.9)	84 (36.8)	179 (38.9)
SBP ≥130 and/or DBP ≥80 (%)	38 (16.4)	34 (14.9)	72 (15.6)
SBP ≥140 and/or DBP ≥90 (%)	73 (31.5)	85 (37.3)	158 (34.4)
Missing, *n* (%)	26 (11.2)	25 (11.0)	51 (11.1)
**LDL-C (mmol/L)**			
<1.8 *n* (%)	9 (21.1)	73 (32.0)	122 (26.5)
≥1.8 *n* (%)	75 (32.3)	92 (40.4)	167 (36.3)
Missing, *n* (%)	108 (46.6)	63 (27.6)	171 (37.2)
Median (IQR)	2.00 (1.03)	1.90 (1.30)	2.00 (1.21)
Mean (SD)	2.26 (0.86)	2.19 (1.02)	2.21 (0.95)
**Non-HDL-C (mmol/L)**			
<2.59 mmol/L *n* (%)	53 (22.8)	85 (37.3)	138 (30.0)
≥2.59 mmol/L *n* (%)	73 (31.5)	79 (34.6)	152 (33.0)
Missing, *n* (%)	106 (45.7)	64 (28.1)	170 (37.0)
Median (IQR)	2.68 (1.14)	2.54 (1.48)	2.61 (1.33)
Mean (SD)	2.95 (0.95)	2.82 (1.16)	2.88 (1.08)
**Triglycerides (mmol/L)**			
<1.7	74 (31.9)	108 (47.4)	182 (39.6)
≥1.7	56 (24.1)	58 (25.4)	114 (24.8)
Missing, *n* (%)	102 (44.0)	62 (27.2)	164 (36.6)
Median (IQR)	1.51 (1.09)	1.40 (0.99)	1.41 (1.04)
Mean (SD)	1.64 (0.74)	1.66 (1.04)	1.66 (0.92)
**Serum potassium (mmol/L)**			
<5.0	138 (59.5)	168 (73.7)	306 (66.5)
≥5.0	14 (6.0)	20 (8.8)	34 (7.4)
Missing, *n* (%)	80 (34.5)	40 (17.5)	120 (26.1)
Median (IQR)	4.4 (0.5)	4.4 (0.7)	4.4 (0.6)
Mean (SD)	4.5 (0.5)	4.4 (0.5)	4.3 (0.5)

### Adherence to the MAT_STEMI_ Criteria

Table 1 shows adherence to the MAT_STEMI_ criteria. MAT_STEMI_ was applied on 232 patients’ medication records, with 3,712 criteria. Of these, 1,658 (52.3%) were labeled “NA” because the patients did not meet the qualifier and were not eligible for the application of the criterion. Hence, 2,054 criteria were assessed; 194 (9.4%) were labeled “IDQ” due to a lack of information on the qualifier to assess whether a criterion was applicable or not. Hence, 1860 (90.6%) criteria were found to be applicable. Of these, 107 (5.8%) were labeled “IDS” due to a lack of information on the standard to assess whether the applicable criterion met the standard statement or not. The overall percentage data gap for the applicable criteria was 5.8% (95% CI: 4.8–6.9). Nine criteria out of 16 achieved high adherence (>80%). Overall adherence was deemed as intermediate adherence (69.8%; 95% CI: 67.6–72.0). Overall unjustified non-adherence was 24.1% (95% CI: 22.1–26.2), and justified non-adherence was 5.2% (95% CI: 4.2–6.3). Criterion 4 had very low applicability (less than 10 patients) and was therefore not included in the calculation of the overall adherence and non-adherence.

### Adherence to the MAT_NSTEACS_ Criteria

Table 2 presents the adherence to the MAT_NSTEACS_ criteria. MAT_**NSTEACS**_ was applied on 228 patients’ medication records with 2,508 criteria. Of these, 1,257 (50.1%) were labeled “NA” because the patients did not meet the qualifier and were not eligible for the application of the criterion. Hence, 1,251 criteria were assessed; 146 (11.7%) were labeled “IDQ” due to a lack of information on the qualifier to assess whether a criterion was applicable or not. Hence, 1,105 (88.3%) criteria were found to be applicable. Of these, 3 (0.3%) were labeled “IDS” due to a lack of information on the standard to assess whether the applicable criterion met the standard statement or not. The overall percentage of the data gap for the applicable criteria was 0.3% (95% CI: 0.1–0.9). Seven criteria out of 11 achieved high adherence (>80%). Overall adherence was deemed as intermediate adherence (73.3%; 95% CI: 70.5–76.0). Overall unjustified non-adherence was 26.4% (95% CI: 23.7–29.2), while that of justified non-adherence was 5.8% (95% CI: 4.6–7.4). Criterion 2 had very low applicability (less than 10 patients) and was therefore not included in the calculation of the overall adherence and non-adherence.

### Comparisons and Factors Associated With Overall Adjusted Adherence to the MAT_STEMI_ Criteria and MAT_NSTEACS_ Criteria

The difference between the percentages of overall adherence among patients managed post-NSTEACS (73.3%) compared to that managed post-STEMI (69.8%) was not statistically significant. The percentage of the data gap was found to be significantly higher among patients with prior STEMI (6.1%) than those with prior NSTEACS (0.3%), *p* < 0.001. In relation to patients managed post-STEMI, there were no significant differences among percentages of the data gap for the applicable criteria between the general hospitals (6.0%) and specialized cardiac centers (5.6%), *p* = 0.79. A similar non-significant difference in the percentages of the data gap for the applicable criteria was found between the general hospitals (0.2%) and specialized cardiac centers (0.4%) for patients managed post-NSTEACS, *p* = 0.94.

When conducting multivariable logistic regression analysis, the results revealed that one variable was significantly associated with the overall adherence to the MAT_STEMI_ criteria. It was significantly higher among the specialized cardiac centers than among general hospitals (OR: 1.6; 95% CI: 1.1–2.3; *p* = 0.02). It was found to be non-significantly higher among the young age-group (20–44 years), male individuals, patients without PMH of stable ischemic heart disease or hypertension, and patients with a BP < 130/80 mmHg or a serum LDL <1.8 mmol/L (*p* > 0.05). The overall adherence to the MAT_NSTE**ACS**_ criteria was found to be significantly associated with nationality and patients’ serum LDL-C levels. It was found to be significantly lower among non-Kuwaitis than among Kuwaitis (OR: 0.6; 95% CI: 0.5–0.9; *p* = 0.01) and patients with a serum LDL ≥1.8 mmol/L than those with serum LDL-C < 1.8 mmol/L (OR: 0.5; 95% CI: 0.4–0.7; *p* < 0.001). It was found to be non-significantly lower among general hospitals, young age-group (20–44 years), male individuals, and patients with PMH of hypertension (*p* > 0.05). Table 5 shows the results of the multivariable analysis for factors associated with overall adherence to the MAT_STEMI_ criteria and MAT_NSTEACS_ criteria. The detailed analysis of the independent variables (health-care facilities and nationality) that revealed a significant association in the multivariable regression analysis showed the following: 1) In relation to the MAT_STEMI_ criteria, it was found that adherence to criteria 1,3,7,8, and 9 was significantly higher among the specialized cardiac centers than among the general hospitals, and 2) regarding the MAT_NSTEACS_ criteria, it was found that the adherence to criteria 5, 7, and 8 was significantly lower among non-Kuwaitis than among Kuwaitis.

**TABLE 5:** Association between the overall adherence to the MAT_STEMI_ criteria and MAT_NSTEACS_ criteria and patients’ characteristics.

Characteristic	MAT_STEMI_ criteria		MAT_NSTEACS_ criteria	
	OR (95% CI)	*p*-value	OR (95% CI)	*p*-value
**Health-care facilities**		0.02*		0.27
General hospitals	Reference		Reference	
Specialized cardiac centers	1.6 (1.1–2.3)		1.2 (0.9–1.7)	
**Age (years)**		0.81		0.40
20–44	Reference		Reference	
45–64	0.8 (0.4–1.7)		1.4 (0.8–2.4)	
≥65	0.8 (0.3–1.7)		1.3 (0.7–2.3)	
**Sex**		0.78		0.34
Male	Reference		Reference	
Female	0.9 (0.6–1.6)		1.2 (0.8–1.7)	
**Nationality**		0.21		
Kuwaiti	Reference		Reference	0.01*
Non-Kuwaiti	0.7 (0.5–1.2)		0.6 (0.5–0.9)	
**PMH of stable ischemic heart disease**		0.63		-
No	Reference		-	
Yes	0.8 (0.7–1.7)		-	
**PMH of hypertension**		0.83		0.30
No	Reference		Reference	
Yes	0.9 (0.6–1.5)		0.8 (0.6–1.2)	
**BP categories (mmHg)**		0.31		-
SBP and DBP <130/80	Reference		-	
SBP ≥130 and/or DBP ≥80	0.9 (0.5–1.6)		-	
SBP ≥140 and/or DBP ≥90	0.7 (0.5–1.1)		-	
**Serum LDL (mmol/L)**		0.29		<0.001*
<1.8	Reference		Reference	
≥1.8	0.8 (0.5–1.2)		0.5 (0.4–0.7)	

Variables with *p* > 0.25 in the univariable analysis, which were not included in the multivariable analysis.

*p<0.05.

## Discussion

The goal of CPGs is to increase high-quality care and reduce inappropriate interventions (Grimshaw and Russell, 1993). Evidence has shown that there is a gap in the literature regarding the development, implementation, and evaluation of CPGs in developing countries including the MENA region (Koornneef et al., 2015). To the best of our knowledge, this is the first study in the developing countries including the MENA region to comprehensively assess the quality of prescribing practices for secondary prevention of CHD in post-STEMI and post-NSTEACS patients attending secondary and tertiary health-care settings. The present findings can be used for planning and evaluating any future interventions using these MATs as outcome measures and allowing for vital comparative work with prevailing and future similar research in the MENA countries and worldwide.

The present findings showed an overall adherence of 69.8% and 73.3% to post-STEMI and post-NSTEACS ESC and ACC/AHA CPGs, respectively. These are higher than the adherence scores observed in our pilot study (64.1% and 62.0% for MAT_STEMI_ and MAT_NSTEACS,_ criteria, respectively) (Al-Taweel and Awad, 2020). The present overall adherence scores to both MATs were judged as intermediate, which are comparable to those reported in earlier studies that used the MAT methodology in developed countries to audit the secondary prevention of CHD (Kamyar et al., 2008; Garcia et al., 2013; Garcia et al., 2011). This intermediate overall adherence may be explained by the lack of locally generated evidence-based guidelines for the management of CVDs, as well as the limited multidisciplinary approach to the management of patients with CVDs in Kuwait, which makes it more difficult to adapt clinical standards into practice (Laing et al., 2001).

Applicability to our MAT was found to be 90.6% for post-STEMI patients and 88.3% for post-NSTEACS patients. This is higher than that reported in previous studies that used the MAT methodology (Hakonsen et al., 2006; Kamyar et al., 2008; Hakonsen et al., 2009; Al-Taweel et al., 2013; Diab et al., 2013; Garcia et al., 2013; Al-Taweel and Alsuwaidan, 2017). One criterion in MAT_STEMI_ (the patient post-fibrinolysis, without subsequent PCI, is prescribed clopidogrel 75 mg daily in addition to aspirin for at least 14 days and up to 1 year in absence of bleeding) and one in MAT_NSTEACS_ (the patient who is not prescribed aspirin due to hypersensitivity is prescribed clopidogrel 75 mg daily) were found to have very low applicability (less than 10 patients) and were therefore not accounted for when calculating adherence to give a more accurate evaluation. However, these criteria were not deleted from the tools as excluding them could omit important aspects from cardiovascular medication management in a small number of eligible patients. This is consistent with Garcia et al.’s study when assessing adherence to guidelines in post-PCI patients as they stated “inclusion and exclusion of criteria do not exclusively concern applicability but also the clinical relevance” (Garcia et al., 2013). Moreover, our study showed that IDS and IDQ had relatively low frequency, which could reduce the bias potential. The MATs used in this study have the advantage of offering an opportunity to quantify the frequency of justified deviations from CPG standards and also to detect inappropriate documentation in the patient medical records, which again may assist in retrospectively assessing the appropriateness of patient care.

The present study population had many comorbidities, including stable IHD, hypertension, diabetes, and dyslipidemia. Adherence to standards related to antiplatelets, beta-blockers, and angiotensin-converting enzyme inhibitors (ACEIs) or angiotensin II receptor blockers (ARBs) was found to be high (>80%) in the management of both post-STEMI and post-NSTEACS patients; however, adherence to lipid-lowering therapy was intermediate to low. This is consistent with the results of a previous study which showed that among 6 Middle Eastern countries, Kuwait was found to have the lowest utilization of statins among these countries. (Al-Zakwani et al., 2011). A possible explanation is that the physicians were more preoccupied with the potential side effects of statins. Other possible reasons might be statin intolerance, lack of acceptance of CPGs’ recommendations, and contraindications (Boklage et al., 2018)*.* However, the main drivers for suboptimal prescribing adherence to statins post-ACS need further examination. Evidence has shown that antiplatelet agents, beta-blockers, ACEIs/ARBs, and lipid-lowering agents (called the “quadruple regimen”) are individually efficient in lowering secondary cardiovascular events, and when combined, they are even more effective in preventing cardiac events (Melloni and Newby, 2009). The utilization of clopidogrel post-PCI in this study was found to be higher than that reported in Kuwait (Al-Zakwani et al., 2011), which indicates an improvement in adapting the evidence-based CPG recommendations regarding dual antiplatelet therapy.

This study revealed incomplete documentation of patients’ records, which is consistent with a previous study using the MAT methodology but on a different disease state (Al-Taweel and Alsuwaidan, 2017). This may be out of habit or due to the heavy workload and time constraints. Most physicians might spend their limited amount of time providing patient care, and documentation becomes the secondary priority. This finding highlights the need for a further qualitative study to determine the factors causing this incomplete documentation of patients’ records. MATs continue to prove to be valuable tools in revealing gaps in documentation practice. Documentation gaps occurred mostly due to incomplete entry of patients’ laboratory results, which results in discontinuity of care between health-care teams, and could lead to a low estimate of adherence and do not necessarily indicate a lack of care. The difference between the documentation gap in patients’ records post-STEMI compared to that in patients post-NSTEACS is a cause for concern and may need to be studied further. The use of electronic medical records could be a solution to improve documentation and ultimately improve decision-making (Gill et al., 2009). However, they seem to be underutilized with many data fields left blank. A recent study conducted in Kuwait revealed that many physicians reported organizational, technological, and economic factors as barriers to electronic medical record use. They also identified other barriers including lack of electronic health record awareness, lack of computer skills and experience, and loss of personal attention with the patient while entering data (Alaslawi et al., 2019).

The present findings showed low achievement of optimal therapy goals for BP (43.8%), LDL-C (42.2%), and non-HDL-C (47.6%) among the study population. This is alarming and increases the risk for recurrent cardiovascular events. It has to be emphasized that successful management of CVDs involves not only the treatment of a specific disease but also treating and preventing the risk factors for CVD, including diabetes, dyslipidemia, and hypertension (Indio do Brasil et al., 2013). Limitations to patients achieving target goals reported in the literature include inappropriate prescribing practices, poor patient adherence to medication, and unawareness of the importance of therapeutic goal attainment (Whitley et al., 2006). Our results could serve as an initial step in providing baseline quantitative data of patients who have not reached their targets along with adherence scores, and that can be used for planning and evaluating any future interventions or initiatives to improve target achievement in this critical patient group.

Our data included patients from both general hospitals (secondary care) and specialized cardiac hospitals (tertiary care). Multivariable logistic regression analysis revealed that the one variable that was significantly associated with the overall adherence to the MAT_STEMI_ criteria, was the site of care—as specialized cardiac centers (tertiary care) had a significantly higher adherence compared to the general hospitals (OR: 1.6; 95% CI: 1.1–2.3; *p* = 0.02). The overall adherence to the MAT_NSTEACS_ criteria was found to be non-significantly higher among specialized care centers than among general hospitals (OR: 1.2; 95% CI: 0.9–1.7). A previous study in the Canadian registry revealed that patients with STEMI were more likely to receive the evidence-based post-acute coronary syndrome therapies compared with patients with NSTEACS (Yan et al., 2007), which could be a possible explanation for this non-significant difference in overall adherence to the MAT_NSTEACS_ criteria between the specialized care centers and the general hospitals. The present findings are consistent with those of a previous study which found out that the use of evidence-based medications in patients with cardiovascular disorders was more likely to be associated with patients who had cardiologists as care providers (Al-Zakwani et al., 2011), which is the case in tertiary care settings in Kuwait, where as in secondary care settings physicians could be internists. In addition, with the growing management complexity of cardiovascular disorders, it has been proven that the benefits of subspecialty care on those patients with ACS far outweigh general care and results in the decreasing length of stay in hospitals, cardiovascular readmission, and mortality (Pathik et al., 2015). The overall adherence to the MAT_NSTEACS_ criteria was found to be significantly lower among non-Kuwaitis than among Kuwaitis (OR: 0.6; 95% CI: 0.5–0.9; *p* = 0.01) and patients with a serum LDL-C ≥1.8 mmol/L than those with serum LDL-C < 1.8 mmol/L (OR: 0.5; 95% CI: 0.4–0.7; *p* < 0.001). The overall adherence to the MAT_STEMI_ criteria was found to be non-significantly lower among non-Kuwaitis than among Kuwaitis (OR: 0.7; 95% CI: 0.5–1.2; *p* = 0.63) and patients with a serum LDL-C ≥ 1.8 mmol/L than those with a serum LDL-C < 1.8 mmol/L (OR: 0.8; 95% CI: 0.5–1.2; *p* = 0.29). Further qualitative studies are required to allow a comprehensive understanding of the factors associated with the overall adherence to the MAT_STEMI_ and MAT_NSTEACS_ criteria.

## Strengths and Limitations

This study has several strengths: 1) the validated MAT_STEMI_ and MAT_NSTEACS_ would be useful in routine clinical practice to provide feedback to the physicians, which identify aspects of care, that are to be prioritized for improvement; 2) the appropriate sample size and sampling methods to yield representative data about the study population; thus, the current results can be generalized to post-STEMI and post-NSTEACS patients attending secondary and tertiary health-care settings in Kuwait; and 3) it adds to the limited literature on prescribing practices in the management of cardiovascular disorders in developing countries including the MENA region and allows for important comparative work with future similar studies in the region and worldwide.

There were certain limitations in the current study, which include the following: 1) The retrospective nature of the study that depends on the quality of documentation in the patient medical records and thus may not provide the true adherence of prescribers to CPGs. However, our study did take that into account by quantifying “insufficient data” and reporting the % data gap; 2) the duration of time for application of the MAT per medication record was not measured, and this would aid in supporting the clinical utility of the MATs in a real-world setting; and 3) the present study did not assess the impact of the prescribing adherence rates on clinical outcomes (e.g., stable disease, relapse, and death), which would have been highly relevant additional findings.

## Conclusions

The MAT_STEMI_ and MAT_NSTEACS_ provide a method to identify both adherence and non-adherence to CPGs for secondary prevention of CHD in post-ACS patients and identify potentials for improved patient care. The current results could serve as an initial step in providing baseline quantitative data of prescribing adherence to post-STEMI and post-NSTEACS CPGs and underscore the need for cost-effective multifaceted interventions to improve current prescribing practices and documentation. Future studies are warranted to assess the impact of the adherence rates demonstrated by this study on clinical outcomes such as hospitalization and mortality among post-ACS patients.

## Data Availability

The raw data supporting the conclusions of this article will be made available by the authors, without undue reservation.
